# Genome-wide gene order distances support clustering the gram-positive bacteria

**DOI:** 10.3389/fmicb.2014.00785

**Published:** 2015-01-20

**Authors:** Christopher H. House, Matteo Pellegrini, Sorel T. Fitz-Gibbon

**Affiliations:** ^1^Penn State Astrobiology Research Center and Department of Geosciences, The Pennsylvania State UniversityUniversity Park, PA, USA; ^2^Department of Molecular, Cell, and Developmental Biology, University of California, Los AngelesLos Angeles, CA, USA; ^3^Department of Molecular, Cell, and Developmental Biology, Institute of Genomics and Proteomics, University of California, Los AngelesLos Angeles, CA, USA

**Keywords:** tree of life, gene order, evolutionary distance, genomics, Actinobacteria, Firmicutes, Archaea

## Abstract

Initially using 143 genomes, we developed a method for calculating the pair-wise distance between prokaryotic genomes using a Monte Carlo method to estimate the conservation of gene order. The method was based on repeatedly selecting five or six non-adjacent random orthologs from each of two genomes and determining if the chosen orthologs were in the same order. The raw distances were then corrected for gene order convergence using an adaptation of the Jukes-Cantor model, as well as using the common distance correction D′ = −ln(1-D). First, we compared the distances found via the order of six orthologs to distances found based on ortholog gene content and small subunit rRNA sequences. The Jukes-Cantor gene order distances are reasonably well correlated with the divergence of rRNA (*R*^2^ = 0.24), especially at rRNA Jukes-Cantor distances of less than 0.2 (*R*^2^ = 0.52). Gene content is only weakly correlated with rRNA divergence (*R*^2^ = 0.04) over all distances, however, it is especially strongly correlated at rRNA Jukes-Cantor distances of less than 0.1 (*R*^2^ = 0.67). This initial work suggests that gene order may be useful in conjunction with other methods to help understand the relatedness of genomes. Using the gene order distances in 143 genomes, the relations of prokaryotes were studied using neighbor joining and agreement subtrees. We then repeated our study of the relations of prokaryotes using gene order in 172 complete genomes better representing a wider-diversity of prokaryotes. Consistently, our trees show the Actinobacteria as a sister group to the bulk of the Firmicutes. In fact, the robustness of gene order support was found to be considerably greater for uniting these two phyla than for uniting any of the proteobacterial classes together. The results are supportive of the idea that Actinobacteria and Firmicutes are closely related, which in turn implies a single origin for the gram-positive cell.

## Introduction

For the past three decades, the comparisons of ribosomal RNA (rRNA) between microorganisms have largely provided the taxonomic and phylogenetic basis for bacteriology (Woese, 1987). During the past 15 years, however, considerable effort has been placed on comparing the similarity of organisms with genome-wide methods or, at least, with methods that use more than a single gene. These methods include the estimation of genomic distances based on the content of genomes, either orthologs, homologs, folds, or protein domains (Gerstein, 1998; Fitz-Gibbon and House, 1999; Snel et al., 1999; Tekaia et al., 1999; Wolf et al., 2002; Deeds et al., 2005; Yang et al., 2005; House, 2009). Genomic distance has also been estimated using direct genome-to-genome sequence comparisons using a variety of approaches like average nucleotide identity (ANI) and the genome-to-genome-distance calculator (GGDC) that can approximate traditional DNA-DNA hybridization results (Konstantinidis and Tiedje, 2005; Goris et al., 2007; Deloger et al., 2009; Richter and Rosselló-Móra, 2009; Auch et al., 2010; Tamura et al., 2012; Meier-Kolthoff et al., 2013). Also, ever since Nadeau and Taylor (1984) first identified that gene order information was conserved between humans and mice, there has been growing interest in using gene order to estimate the difference between genomes or to solve phylogenetic problems.

Several gene order methods depend on the presence of orthologs adjacent to each other. Watterson et al. (1982) introduced the *breakpoint distance* between genomes, which is the number of orthologs found paired together in one genome but separated in the other Blanchette et al. (1999). Early on, Sankoff et al. (1992) estimated mitochondria gene rearrangements as a means to derive a phylogenetic tree for Eukaryotes. Subsequently, the presence and absence of paired genes has been used to construct trees (Wolf et al., 2001; Korbel et al., 2002) as a gene order method similar in practice to tree building by gene content. A limitation to this approach results from the fact that small groups of laterally transferred genes will be paired after their transfer. Also, a computational method for testing phylogenetic problems using gene order has been presented by Kunisawa (2001). In this method, genomes are searched for cases in which the arrangement of three genes most parsimoniously suggests that a single transposition has occurred. With the use of an outgroup, the method can be used to test phylogenetic hypotheses, such as the branching order within the Proteobacteria (Kunisawa, 2001) or Gram-positive bacteria (Kunisawa, 2003). The strength of this method is that it can be efficiently applied to a large dataset of genomes and that it reveals (a small number of) interesting cases of transposition. Another gene order approach often implemented is calculating the inversion distance. The *inversion distance* is the minimum possible number of inversions needed to transform one genome into the other (Moret et al., 2001). Recently, Belda et al. (2005) have studied a subset of 244 genes universal to the genomes of 30 γ-Preotobacteria using both the breakpoint distance and the inversion distance. They found the two distances highly correlated suggesting that inversion was the main method of genome rearrangement for these taxa. More recently, models for genome evolution that include rearrangements, duplications, and losses have been developed and tested (Swenson et al., 2008; Zhang et al., 2010; Hu et al., 2011; Lin and Moret, 2011; Shao et al., 2013) have each developed algorithms for using gene order for phylogenetic reconstruction. Furthermore, Lin et al. (2013) and Shifman et al. (2014) have used genome-wide gene order to produce phylogenetic trees. The later work produced a tree of 89 diverse microbial genomes using an algorithm for estimating average genome synteny (Shifman et al., 2014).

In this study, we aimed to develop a simple computational method that could estimate a genome-wide gene order distance between two genomes (even when the genomes were highly diverged). Unlike many previous efforts, our intent was to have the gene order distance not rely on genes that are likely to be in the same operon (such as gene pairs). Here, we present a novel simple Monte Carlo method for estimating *distributed gene order distances* between genomes. In this method, we repeatedly randomly select six non-adjacent orthologs from each of two genomes and determine if the genes are in the same order. The distances are then corrected using an adaptation of the Jukes-Cantor model to account for random gene order convergence.

## Materials and methods

Initially, 143 prokaryotic genomes were analyzed (Table 1). This represented completed prokaryotic genomes available when the study began in January 2005. All genes from each genome were analyzed as queries using BLAST against each of the other genomes. Ortholog-pairs were identified as cases where two genes from different genomes were each other's BLAST best hit (top hit in both directions). This list of ortholog pairs served as the basis for both calculation of distributed gene order distances and the ortholog gene content distances. As defined by Snel et al. (1999), ortholog gene content similarity (S) was calculated as the number of ortholog pairs found for two genomes divided by the size of the smaller genome. This similarity was then converted to distance as equal to –ln(S), as suggested by Korbel et al. (2002). However, using distance equal to 1-S gives similar correlation results.

**Table 1:** **143 taxa**.

	**ID**
Aeropyrum pernix K1	ap
Agrobacterium tumefaciens C58UW	at
Agrobacterium tumefaciens C58C	atc
Aquifex aeolicus VF5	aa
Archaeoglobus fulgidus DSM4304	af
Bacillus anthracis Ames	baa
Bacillus cereus ATCC 14579	bc
Bacillus halodurans C-125	bh
Bacillus subtilis 168	bs
Bacteroides thetaiotaomicron	bt
Bifidobacterium longum NCC2705	bl
Bordetella bronchiseptica	bbr
Bordetella parapertussis	bpp
Bordetella pertussis	bp
Borrelia burgdorferi B31	bb
Bradyrhizobium japonicum USDA 110	bj
Brucella melitensis	bm
Brucella suis	brs
Buchnera aphidicola Bp	ba
Buchnera aphidicola Sg	bas
*Buchnera* sp. APS	bu
Campylobacter jejuni NCTC 11168	cj
Candidatus Blochmannia floridanus	cbf
Caulobacter crescentus CB15	cc
Chlamydia trachomatis serovar D	ct
Chlamydia trachomatis MoPn/Nigg	cm
Chlamydophila caviae GPIC	cca
Chlamydophila pneumoniae AR39	cpa
Chlamydophila pneumoniae J138	cpj
Chlamydophila pneumoniae TW183	cpt
Chlamydophila pneumoniae CWL029	cp
Chlorobium tepidum TLS	cte
Chromobacterium violaceum	cv
Clostridium acetobutylicum ATCC 824	ca
Clostridium perfringens	cpe
Clostridium tetani	clt
Corynebacterium diphtheria	cd
Corynebacterium efficiens YS-314	cef
Corynebacterium glutamicum	cg
Coxiella burnetii	cb
Deinococcus radiodurans R1	dr
Enterococcus faecalis V583	ef
Escherichia coli O157:H7 strain EDL933	ece
Escherichia coli K-12 Strain MG1655	ec
Escherichia coli CFT073	ecc
Escherichia coli O157:H7	ech
Fusobacterium nucleatum ATCC 25586	fn
Gloeobacter violaceus	gv
Haemophilus ducreyi	hd
Haemophilus influenzae Rd KW20	hi
*Halobacterium* sp. NRC-1	hsp
Helicobacter hepaticus ATCC 51449	hh
Helicobacter pylori 26695	hp
Helicobacter pylori J99	hpj
Lactobacillus plantarum WCFS1	lp
Lactococcus lactis IL1403	ll
Leptospira interrogans s.l. 56601	li
Listeria innocua clip11262	lin
Listeria monocytogenes EGD-e	lm
Mesorhizobium loti MAFF303099	ml
Methanobacterium thermoautotroph.	mt
Methanococcus jannaschii DSM 2661	mj
Methanopyrus kandleri AV19	mk
Methanosarcina acetivorans C2A	ma
Methanosarcina mazei Goe1	mma
Mycobacterium bovis bovis	mb
Mycobacterium leprae	mle
Mycobacterium tuberculosis H37Rv	mtb
Mycobacterium tuberculosis cdc1551	mtc
Mycoplasma gallisepticum	mga
Mycoplasma genitalium G-37	mg
Mycoplasma penetrans	mpe
Mycoplasma pneumoniae M129	mp
Mycoplasma pulmonis UAB CTIP	mpu
Nanobacterium equitans Kin4-M	neq
Neisseria meningitidis MC58	nmm
Neisseria meningitidis A Z2491	nmz
Nitrosomonas europaea	ne
*Nostoc* sp. PCC7120	ns
Oceanobacillus iheyensis HTE831	oi
Pasteurella multocida Pm70	pm
Photorhabdus luminescens	pl
Pirellula_sp	pi
Porphyromonas gingivalis	pg
Prochlorococcus marinus CCMP1375	pmc
Prochlorococcus marinus MED4	pmm
Prochlorococcus marinus MIT9313	pma
Pseudomonas aeruginosa PAO1	psa
Pseudomonas putida KT2440	psp
Pseudomonas syringae pv. tomato	pss
Pyrobaculum aerophilum IM2	pa
Pyrococcus abyssi	pab
Pyrococcus furiosus DSM3638	pf
Pyrococcus horikoshii OT3	ph
Ralstonia solanacearum	rs
Rickettsia conorii Malish 7	rc
Rickettsia prowazekii Madrid E	rp
Salmonella enterica Typhi	se
Salmonella enterica Typhi_Ty2	set
Salmonella typhimurium LT2	sty
Shewanella oneidensis	so
Shigella flexneri 2a	sf
Sinorhizobium meliloti 1021	sm
Staphylococcus aureus N315	san
Staphylococcus aureus MW2	saw
Staphylococcus aureus Mu50	sam
Staphylococcus epidermidis 12228	sep
Streptococcus agalactiae 2603	sa
Streptococcus agalactiae NEM316	sag
Streptococcus mutans	smu
Streptococcus pneumoniae R6	spn
Streptococcus pneumoniae TIGR4	spt
Streptococcus pyogenes SSI-1	mle
Streptococcus pyogenes MGAS8232	spa
Streptococcus pyogenes MGAS315	spg
Streptococcus pyogenes M1_GAS	spm
Streptomyces avermitilis MA-4680	sav
Streptomyces coelicolor A3(2)	sco
Sulfolobus solfataricusP2	ss
Sulfolobus tokodaii 7	st
*Synechococcus* sp. WH8102	syo
*Synechocystis* sp. PCC 6803	sy
Thermoanaerobacter tengcongensis	tt
Thermoplasma acidophilum	ta
Thermoplasma volcanium GSS1	tv
Thermosynechococcus elongatus BP-1	te
Thermotoga maritima MSB8	tm
Treponema pallidum Nichols	tp
Tropheryma whipplei Twist	tw
Tropheryma whipplei TW08_27	twt
Ureaplasma urealyticum serovar 3	uu
Vibrio cholerae serotype O1 (N16961)	vc
Vibrio parahaemolyticus RIMD 2210633	vp
Vibrio vulnificus CMCP6	vv
Vibrio vulnificus YJ016	vvy
Wigglesworthia brevipalpis	wb
Wolinella_succinogenes	ws
Xanthomonas axonopodis pv citri 306	xa
Xanthomonas campestris ATCC 33913	xc
Xylella fastidiosa 9a5c	xf
Xylella fastidiosa Temecula1	xft
Yersinia pestis CO-92 Biovar Orientalis	yp
Yersinia pestis KIM	ypk

**Figure 1:**
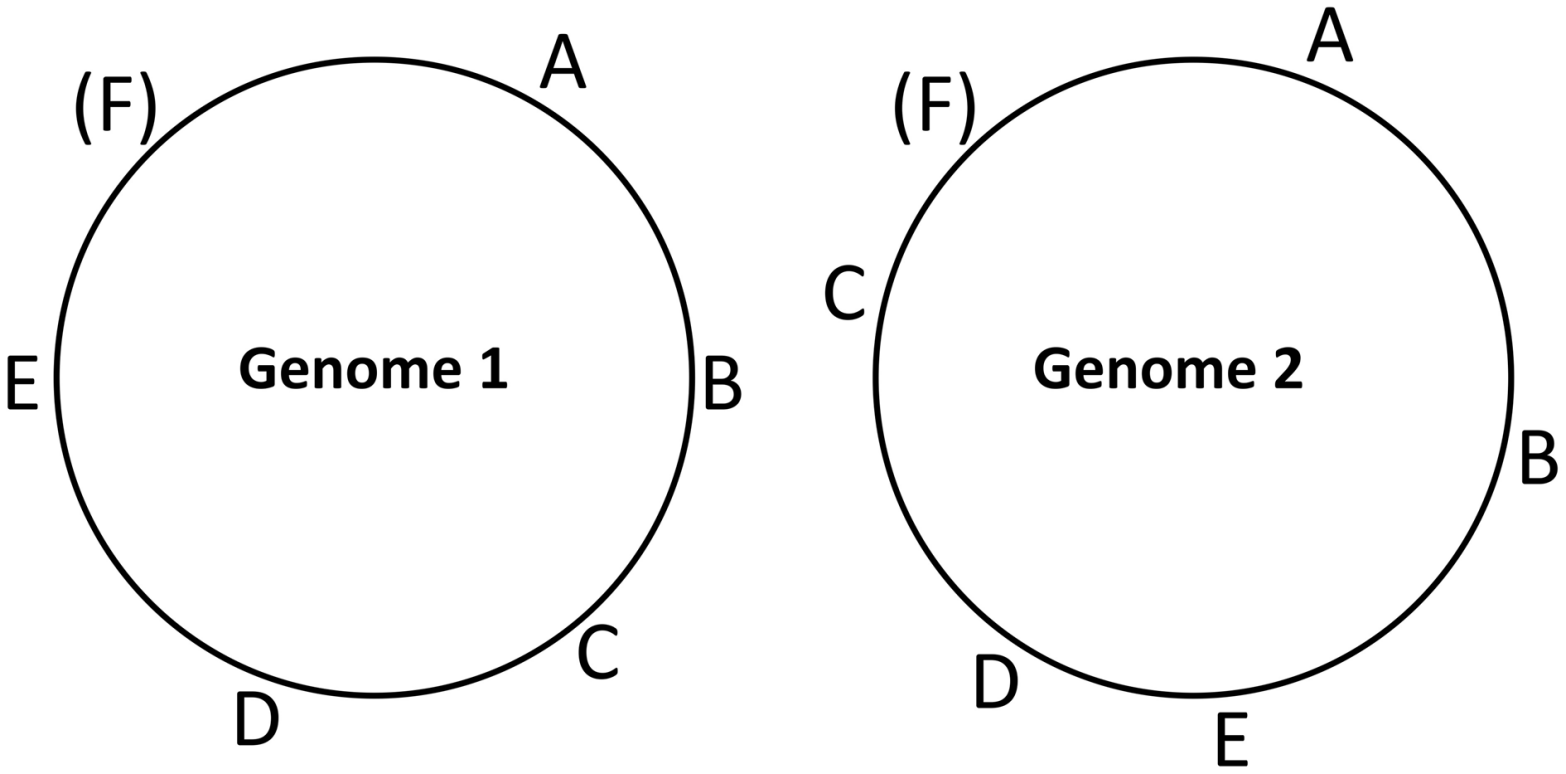
**Diagram demonstrating the method used to calculate the pair wise distributed gene order distance between genomes**. Repeatedly, six ortholog pairs are chosen randomly (requiring every gene in the six be at least 5 genes away along the genome from each). The six genes are then tested to see if they are in the same order (irrespective of the orientation of the genes). In the case above, the test fails because orthologs C and E are switched. Distributed gene order distance is equal to the fraction of times such a test fails between two genomes. The diagram also works for demonstrating the distributed gene order distance between genomes using five genes (A–E) by ignoring gene F.

Distributed gene order distances were determined using a novel Monte Carlo approach (Figure 1). To determine the gene order distance between two genomes, first, six ortholog-pairs were randomly chosen. In order to limit orthologs being chosen from the same operon, the orthologs were required to be at least 5 genes away from each other in either genome. It was then determined if the chosen six ortholog-pairs were in the same order around both circular genomes (irrespective of each genes orientation). For organisms with multiple chromosomes, only the largest chromosome was used in this initial effort. This procedure was repeated for 100,000 iterations to establish one replicate sampling. In the end, 100 replicate samplings were performed for all genome pairs, and these data were either combined to construct one list of distances based on 10 million iterations, or kept separate to make 100 lists of distances for use as bootstrap replicates (nexus files for PAUP are available in Supplementary Material).

Recently diverged genomes begin with close to 100% of their genes arranged in the same order, and with time, the synteny between the genomes decreases. Because there are only 60 different ways to arrange six items on a circle, there is a 1/60 probability of two genomes sharing an arrangement of six orthologs by chance. Therefore, the fraction of six ortholog picks found to be in the same order will ultimately approach 1/60 as divergence time goes to infinity. We, therefore, developed a model of gene order evolution based on the Jukes-Cantor concept that divergence is a logarithmic function with time (Jukes and Cantor, 1969).

The typical Jukes-Cantor correction (Kimura and Ohta, 1972) for nucleotide distance is:

(1) $$D_{JC}=-(3/4)\ln(1-(4/3)D)$$

where *D* = the observed fractional of nucleotides found to be different between two compared genes.

This classical nucleotide Jukes-Cantor correction (Equation 1) accounts for back substitution and is based on a model in which the outcome of any nucleotide substitution can be one of three possibilities. To adopt this logic to gene rearrangements, the Jukes-Cantor equation becomes:

(2) $$D_{JC}=-(59/60)\ln(1-(60/59)D)$$

where *D* = the fraction of iterations in which the six ortholog-pairs chosen are not in the same order.

The classical Jukes-Cantor nucleotide correction (Equation 1) can only be used for raw D up to 0.75. With raw nucleotide distances greater than 0.75, the argument of the logarithm will be zero. To use data in which D is larger than 0.75, Tajima (1993) presented a method using a Taylor series expansion to avoid the logarithm. In our case, Equation (2) fails whenever the raw D is greater than 59/60 (or 0.983). To allow corrections for all of our genome pair distances, we have adopted the method of Tajima (1993) as follows:

(3) $$D_{JC}=\sum_{i\;=\;1}^{k}\frac{k^{\left(i\right)}}{i{\left(59/60\right)}^{i-1}n^{\left(i\right)}}$$

where *k*^(*i*)^ = *k*!/(*k* − *i*)!, *n*^(*i*)^ = *n*!/(*n* − *i*)!, *k* = the number of times the six orthologs are not in the same order, and *n* = the number of iterations used.

Partial reanalysis of the work reported here demonstrated the results are similar when applying D′ = −ln(1-D) as the distance correction rather than the Tajima correction (data not shown), and further future work evaluating this measure of gene order distance is warranted as it is computationally much less intense.

For comparison, Jukes-Cantor corrected rRNA distances were downloaded spring 2006 from the ribosomal database (Cole et al., 2007). The correlations between distributed gene order, gene content, and rRNA distances were performed with SPSS 13 (SPSS, Inc. Chicago, IL) for Mac OS X. Taxonomic assignments for taxa were from the NCBI taxonomic server (Bischoff et al., 2007).

Our follow-up analysis used 172 complete genomes with the aim of being a representative sample of prokaryotes. For this follow-on analysis initiated early in 2014, we used ortholog predictions from the OMA website (Dessimoz et al., 2005). This OMA database is continually updated and includes all chromosomes for each microorganism. The updated analysis here of 172 taxa was done with orthologs downloaded in early 2014. In this case, we also tried searching for five orthologs in the same order rather than six using the same equations, which naturally produces slightly shorter distances overall. In fact, the five gene distances used this last analysis are functionally the same as using the easier to calculate D′ = −ln(1-D). Based on the promising results here, we recommend this simpler distance calculation for future work.

Neighbor Joining (NJ) trees (Saitou and Nei, 1987) were created from data matrices using PAUP 4.0b (Mac and Unix versions; Sinauer Associates, Sunderland, MA). Later, agreement subtrees, which identifies the largest possible pruned tree that is consistent within a set of trees, was used to limit the taxa list in order to minimize possible adverse effects of including genome pairs with very little or no gene order conservation. The agreement subtrees were identified using PAUP 4.0b (Mac) based on a comparison of all of our NJ trees produced from the 100 replicate distances.

We also tried using a hierarchical and iterative approach to produce a series of trees (Table 2). This novel method was based on the fact that shorter distances are known with higher confidence than greater distances. The goal of this method of tree building is to provide a systematic and objective way to build a tree that includes as many of the pair wise gene order distances as possible without letting very distant (random) pairs adversely influence the observed phylogenetic positions of the more closely related taxa. We started with a list of genome pairs ranked from shortest to largest gene order distance (available in Supplemental Data). Starting at the top of the list, we moved down the list adding each pair to our working group until enough pair wise distances were included to allow for one or more NJ trees to be built. This process was continued until we had an exhaustive ranked list of possible unrooted NJ trees starting with the top few very closely related taxa and ending ultimately with a NJ tree of all 143 taxa. Moving down the ranked tree list, we evaluated each tree. A tree (unrooted) was rejected if it was found to be incongruent with an earlier unrooted tree. Congruent trees were pared down in number by removing trees that were fully encompassed by another tree and by combining pairs of compatible trees. Trees were combined by building a new NJ tree with the union set of taxa from the two original trees. The trees were only considered compatible for combining if the process did not cause a disruption of either of the original backbone topologies. For each kept tree, we recorded both the rank of the taxa pair that resulted in its initial formation, and the rank of the last taxa pair added. The largest resulting tree (with 37 taxa) was selected for further study. Additional taxa were added using a process of single taxon addition. In this second round of analysis, moving down the ranked list of genome pairs, we attempted to sequentially add additional taxa to the tree. If the addition of the single taxon disrupted the existing NJ topology, then the taxon was not added.

**Table 2:** **Steps used in hierarchical tree building**.

1	Construct a ranked list of gene order distances starting with the shortest distances
2	Move down ranked list, forming NJ trees of increasing taxa number estimating reaching a NJ tree of all taxa
3	In turn, evaluate each tree formed starting with the smallest and moving to the largest
4	Keep trees consistent with all previously retained trees, while rejecting any new tree that is incongruent with a previously retained tree
5	Starting with those represented by the smallest gene order pairs, single taxa were added to the largest retained tree if their addition did not disrupt the existing NJ topology (second round of taxa addition)

## Results and discussion

### Initial test of gene order as an evolutionary distance

**Figure 2:**
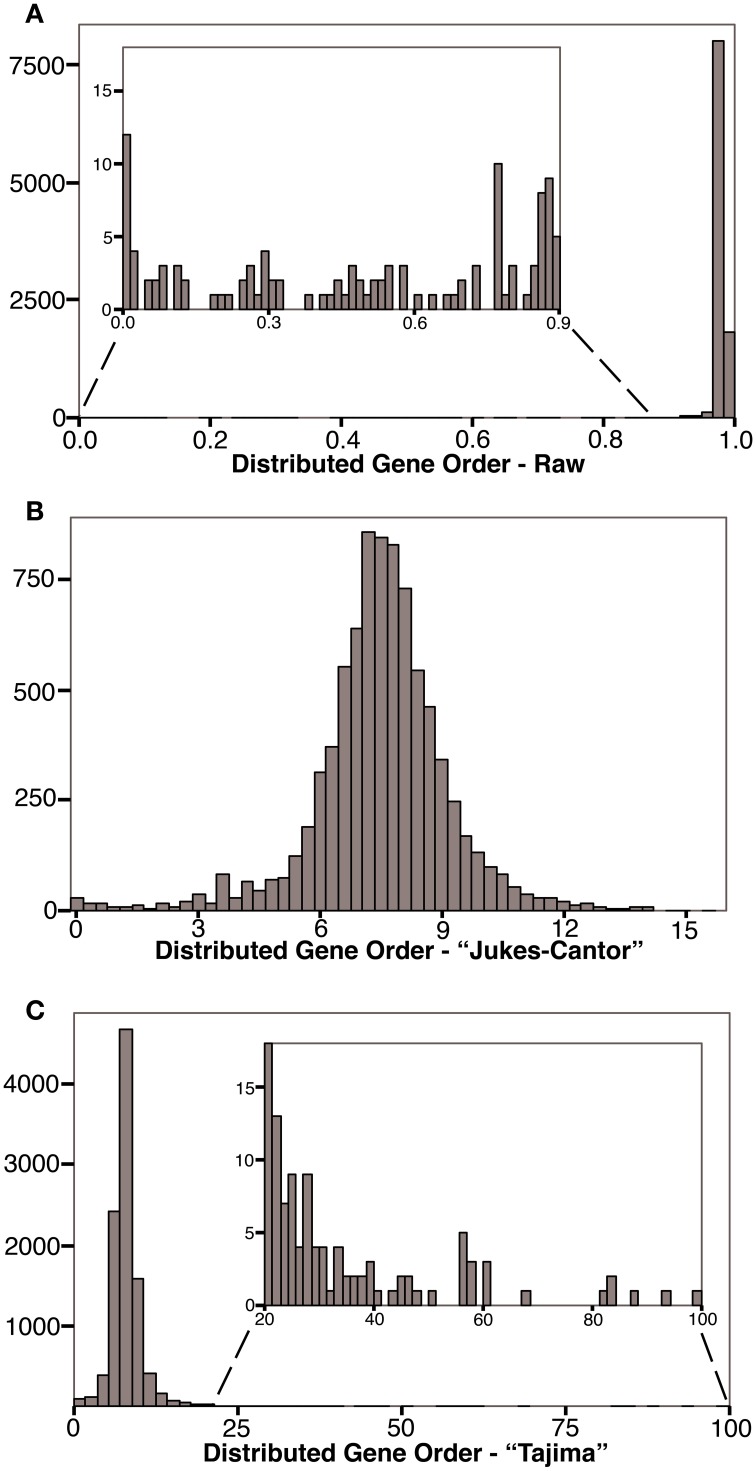
**Histograms showing the frequency of gene order distances calculated for 143 prokaryotes**. **(A)** Distribution of raw gene order distances. The predicted distance for randomly ordered genomes is 0.983, 82% of the genome pairs have raw distances less the 0.983. **(B)** Distribution of distances after a Jukes-Cantor type correction. The predicted “Jukes-Cantor” gene order distance for randomly ordered genomes is >16. Some highly distant genome pairs are not shown in **(B)** because this logarithmic correction cannot be applied to distances greater than that expected randomly. **(C)** Distribution of Tajima-corrected gene order distances. Highly distant genome pairs are extreme outliners due to large corrections applied. Without these genome-pairs, the distribution is similar to that shown in **(B)**.

The distribution of raw gene order distances for each of the 10,153 genome pairs for our 143 genomes are plotted in Figure 2A (and available in Supplementary Material). As expected, with raw gene order distance of 0 (or near 0), the two genomes for *Chlamydia trachomatis*, and separately the four genomes for *Chlamydophila pneumoniae* define the far left of the distribution. The bulk of the genome pairs, however, show raw gene order distances of greater than 0.9 with a peak near, but below, the value expected randomly (0.983). 82% of the genome pairs have gene order distances below 0.983. Figure 2B shows the same data after an adapted Jukes-Cantor model correction (Equation 2). Using this logarithm–based correction, the gene order distances show a relatively normal distribution with a mean of 7.49 (*SD* = 1.68). This correction, however, is not possible for raw gene order distance larger than 0.983, and so, such divergent data are missing from Figure 2B. Figure 2C shows a fuller dataset of gene order distances corrected using the method adopted from Tajima (Equation 3). In this case, a very long tail of very large gene order distances is apparent. This tail is caused by large corrections being applied to some dissimilar genome-pairs.

**Figure 3:**
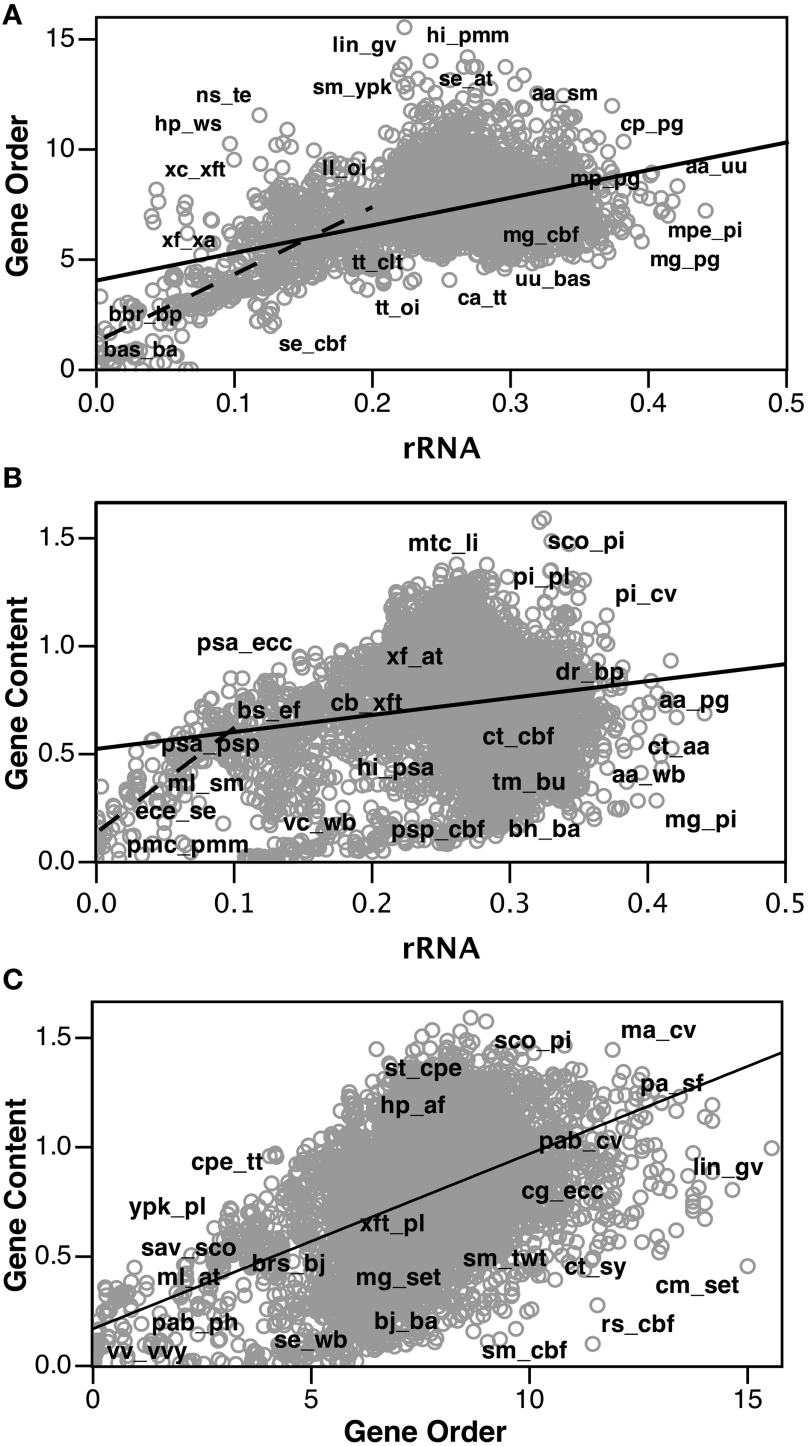
**Comparison of “Jukes-Cantor” distributed gene order distances with ortholog gene content and Jukes-Cantor rRNA distances**. Select gene pairs have been labeled. **(A)** Gene order distance plotted as a function of rRNA distance. Solid line is linear regression of all data (*R*^2^ = 0.24). Dashed line is a linear regression for genome pairs with rRNA distances <0.2 (*R*^2^ = 0.52). **(B)** Gene content distance plotted as a function of rRNA distance. Solid line is linear regression of all data (*R*^2^ = 0.04). Dashed line is linear regression for genome pairs with rRNA distances <0.1 (*R*^2^ = 0.67). **(C)** Gene content distance plotted as a function of gene order distance. Solid line is linear regression of all data (*R*^2^ = 0.22).

After calculating corrected gene order distances for each genome pair, we compared these values with other measures of genome distance, Jukes-Cantor corrected rRNA distances and logarithmic gene content distances (data used are available in Supplementary Material). Figure 3A shows a strong correlation between the “Jukes-Cantor” corrected gene order distances and the Jukes-Cantor rRNA distances (*R*^2^ = 0.24), especially at rRNA distances shorter than 0.2 (*R*^2^ = 0.52). Gene content distances show much less significant correlations with rRNA distance (Figure 3B; *R*^2^ = 0.04), and are actually much more strongly correlated with gene order (Figure 3C; *R*^2^ = 0.22). However, a very strong correlation between gene content and Jukes-Cantor rRNA distance is apparent at rRNA distances shorter than 0.1 (*R*^2^ = 0.67). Apparent in Figure 2B, there is a cluster of genome pairs with similar gene content (low gene content distance) but divergent rRNA sequences (rRNA distances between 0.1 and 0.3 and Gene Content distances of 0.0 and 0.2). This population of genome pairs consists of very small genomes in which extreme genome reduction has occurred. In cases of such extensive genome reduction, the proportion of orthologs shared between two genomes is high even when rRNA sequences indicate that the genomes are relatively distant. With an updated list of genomes, we found our Jukes-Cantor gene order distances were correlated with the divergence of conserved protein genes (*R*^2^ = 0.13 overall and *R*^2^ = 0.23 for cases with less than one amino acid substitution calculated per site). For this comparison, we identified 87 taxa present in both our data (Table 3) and (Lang et al., 2013) and we extracted the aligned amino acid sequences from their published alignment of 841 sequences. Using MEGA5 we computed pairwise distances under the Dayhoff matrix based model (Schwartz and Dayhoff, 1978).

**Table 3:** **172 taxa**.

	**ID**
Acidaminococcus fermentans	ACIFV
Acidilobus saccharovorans	ACIS3
Acidimicrobium ferrooxidans	ACIFD
Acidithiobacillus ferrooxidans	ACIF5
Acinetobacter baumannii	ACIBS
*Acinetobacter* sp.	ACIAD
Aeromonas hydrophila hydrophila	AERHH
Aeromonas salmonicida	AERS4
Alcanivorax borkumensis	ALCBS
Alicyclobacillus acidocaldarius	ALIAD
Alteromonas macleodii	ALTMD
Amycolatopsis mediterranei	AMYMU
Anabaena variabilis	ANAVT
Anoxybacillus flavithermus	ANOFW
Arcanobacterium haemolyticum	ARCHD
Archaeoglobus fulgidus	ARCFU
Archaeoglobus profundus	ARCPA
Archaeoglobus veneficus	ARCVS
*Azoarcus* sp.	AZOSB
Azotobacter vinelandii	AZOVD
Bacillus amyloliquefaciens	BACA2
Bacillus pumilus	BACP2
Bacillus selenitireducens	BACIE
Beutenbergia cavernae	BEUC1
Bifidobacterium adolescentis	BIFAA
Bifidobacterium animalis animalis	BIFAR
Bifidobacterium animalis lactis	BIFA0
Burkholderia mallei	BURMA
Burkholderia thailandensis	BURTA
Campylobacter jejuni HS:41	CAMJC
Campylobacter lari	CAMLR
Catenulispora acidiphila	CATAD
Caulobacter crescentus	CAUCR
Caulobacter segnis	CAUST
Cellvibrio gilvus	CELGA
Cellvibrio japonicus	CELJU
Cenarchaeum symbiosum	CENSY
Clostridium novyi	CLONN
Clostridium perfringens	CLOPS
Clostridium tetani	CLOTE
Coriobacterium glomerans	CORGP
Corynebacterium jeikeium	CORJK
Corynebacterium kroppenstedtii	CORK4
Corynebacterium urealyticum	CORU7
Dechloromonas aromatic	DECAR
Desulfovibrio vulgaris	DESVV
Desulfurococcus kamchatkensis	DESK1
Desulfurococcus mucosus	DESM0
Dichelobacter nodosus	DICNV
Enterobacter cloacae	ENTCS
*Enterobacter* sp.	ENT38
Enterococcus faecalis	ENTFA
Frankia alni	FRAAA
*Frankia* sp.	FRASC
Gardnerella vaginalis	GARV4
Geobacillus kaustophilus	GEOKA
*Geobacillus* sp.	GEOSW
Geobacillus thermodenitrificans	GEOTN
Gloeobacter violaceus	GLOVI
Hahella chejuensis	HAHCH
Halobacterium salinarum	HALSA
Halothermothrix orenii	HALOH
Helicobacter mustelae	HELM1
Helicobacter pylori	HELP5
Hydrogenobacter thermophiles	HYDTT
Kineococcus radiotolerans	KINRD
Korarchaeum cryptofilum	KORCO
Lactobacillus fermentum	LACFC
Lactobacillus helveticus	LACH4
Lactobacillus salivarius	LACSC
Lactococcus lactis cremoris	LACLS
*Lactococcus lactis* subsp. Lactis	LACLA
Legionella pneumophila	LEGPL
Legionella pneumophila pneumophila	LEGPH
Leuconostoc citreum	LEUCK
Leuconostoc gasicomitatum	LEUGT
*Leuconostoc* sp.	LEUS2
Listeria monocytogenes serotype 4b	LISMC
Listeria monocytogenes serovar 1/2a	LISMO
Listeria welshimeri serovar 6b	LISW6
Lysinibacillus sphaericus	LYSSC
*Magnetococcus* sp.	MAGSM
*Methanobacterium* sp.	METSW
Methanocaldococcus fervens	METFA
Methanocaldococcus infernus	METIM
Methanocaldococcus vulcanius	ETVM
Methanocella conradii	METCZ
Methanococcus aeolicus	META3
Methanococcus vannielii	METVS
Methanococcus voltae	METV3
Methanopyrus kandleri	METKA
Methanosaeta concilii	METCG
Methanosaeta harundinacea	METH6
Methanosaeta thermophile	METTP
Methanosarcina acetivorans	METAC
Methanosarcina barkeri	METBF
Methanosarcina mazei	METMA
Methylobacillus flagellates	METFK
Methylococcus capsulatus	METCA
Microcystis aeruginosa	MICAN
Micromonospora aurantiaca	MICAI
*Micromonospora* sp.	MICSL
Moraxella catarrhalis	MORCR
Nanoarchaeum equitans	NANEQ
Natranaerobius thermophiles	NATTJ
Nautilia profundicola	NAUPA
Neisseria meningitides	NEIML
Neisseria meningitidis serogroup B	NEIMG
Nitrosomonas europaea	NITEU
Nitrosomonas eutropha	NITEC
Nitrosopumilus maritimus	NITMS
Nitrososphaera gargensis	NITGG
Nocardia cyriacigeorgica	NOCCG
Nocardia farcinica	NOCFA
*Nocardioides* sp.	NOCSJ
Nostoc azollae	NOSA0
Nostoc punctiforme	NOSP7
*Nostoc* sp.	NOSS1
Oceanobacillus iheyensis	OCEIH
Parvularcula bermudensis	PARBH
Pasteurella multocida	PASMU
Prochlorococcus marinus	PROM4
Prochlorococcus marinus pastoris	PROMP
Propionibacterium acnes	PROAC
Propionibacterium propionicum	PROPF
Pseudomonas fulva	PSEF1
Pseudomonas stutzeri	PSEU5
Psychrobacter arcticus	PSYA2
*Psychrobacter* sp.	PSYWF
Rhizobium etli	RHIEC
Rhizobium meliloti	RHIME
Rhodobacter capsulatus	RHOCB
Rhodobacter sphaeroides	RHOS1
Rhodospirillum centenum	RHOCS
Rhodospirillum rubrum	RHORT
Rickettsia prowazekii	RICPR
Rickettsia typhi	RICTY
Rubrobacter xylanophilus	RUBXD
Saccharomonospora viridis	SACVD
Saccharopolyspora erythraea	SACEN
Sphingomonas wittichii	SPHWW
Staphylococcus carnosus	STACT
Staphylococcus epidermidis	STAES
Staphylococcus lugdunensis	STALH
Streptococcus pyogenes M49	STRPZ
Streptococcus pyogenes M5	STRPG
Streptococcus thermophiles	STRTD
Streptomyces avermitilis	STRAW
Streptomyces coelicolor	STRCO
Streptomyces griseus	STRGG
Streptosporangium roseum	STRRD
Sulfolobus acidocaldarius	SULAC
Sulfolobus islandicus	SULIM
Sulfolobus solfataricus	SULS9
Thermoanaerobacter italicus	THEIA
Thermoanaerobacter mathranii	THEM3
Thermoanaerobacter pseudethanolicus	THEP3
Thermobispora bispora	THEBD
Thermococcus onnurineus	THEON
Thermococcus sibiricus	THESM
*Thermococcus* sp.	THES4
Thermoplasma acidophilum	THEAC
Thermoplasma volcanium	THEVO
Thermoproteus neutrophilus	THENV
Thermoproteus tenax	THETK
Thermoproteus uzoniensis	THEU7
Thiomicrospira crunogena	THICR
Veillonella parvula	VEIPT
Vibrio cholerae serotype O1	VIBCM
Vibrio fischeri	VIBF1
Xanthomonas campestris	XANCP
Xanthomonas oryzae pv. Oryzae	XANOM

In general, these various analyses have shown that the distributed gene order distances are better correlated with sequenced-based distances (both rRNA and conserved proteins) than are gene content distances. Assuming rRNA and protein distances are useful approximations of evolutionary divergence, our results suggest that gene order distance may be useful for taxonomy or phylogenetics in a similar way that genome-to-genome sequence comparison has proven useful as a genomic measure to replace laboratory DNA-DNA hybridization. Both gene content and gene order have their specific issues and so we would not recommend that either genomic method is ever considered the best, but rather that each be used to help inform more traditional molecular sequence analyses when these methods show signal and can be reasonably interpreted. It is also important to note that horizontal gene transfers likely bring in orthologs in the same order, and so gene order does not necessarily avoid complications of integration arising from such transfers, if they are on a large scale.

### Gene order tree building starting from 143 taxa using neighbor joining

**Figure 4:**
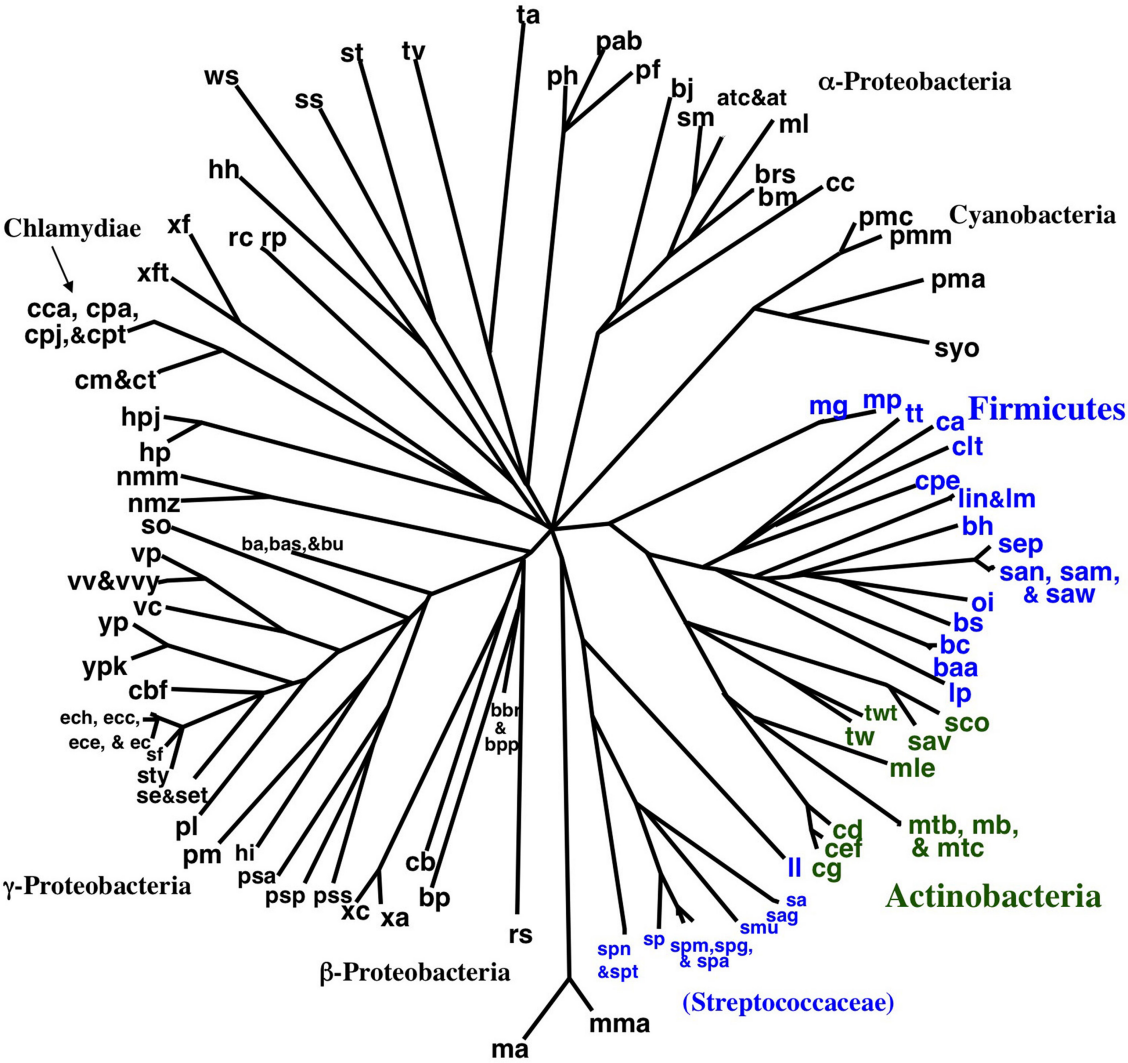
**NJ phylogram of all taxa built using Tajima-corrected gene order distances calculated using 10 million iterations of six predicted orthologs (unresolved single taxon are not shown for clarity)**. Major taxonomic groups are labeled. Actinobacteria are shown in green, and the two clusters of Firmicutes are shown in blue. The Actinobacteria are grouped with the bulk of the Firmicutes.

**Figure 5:**
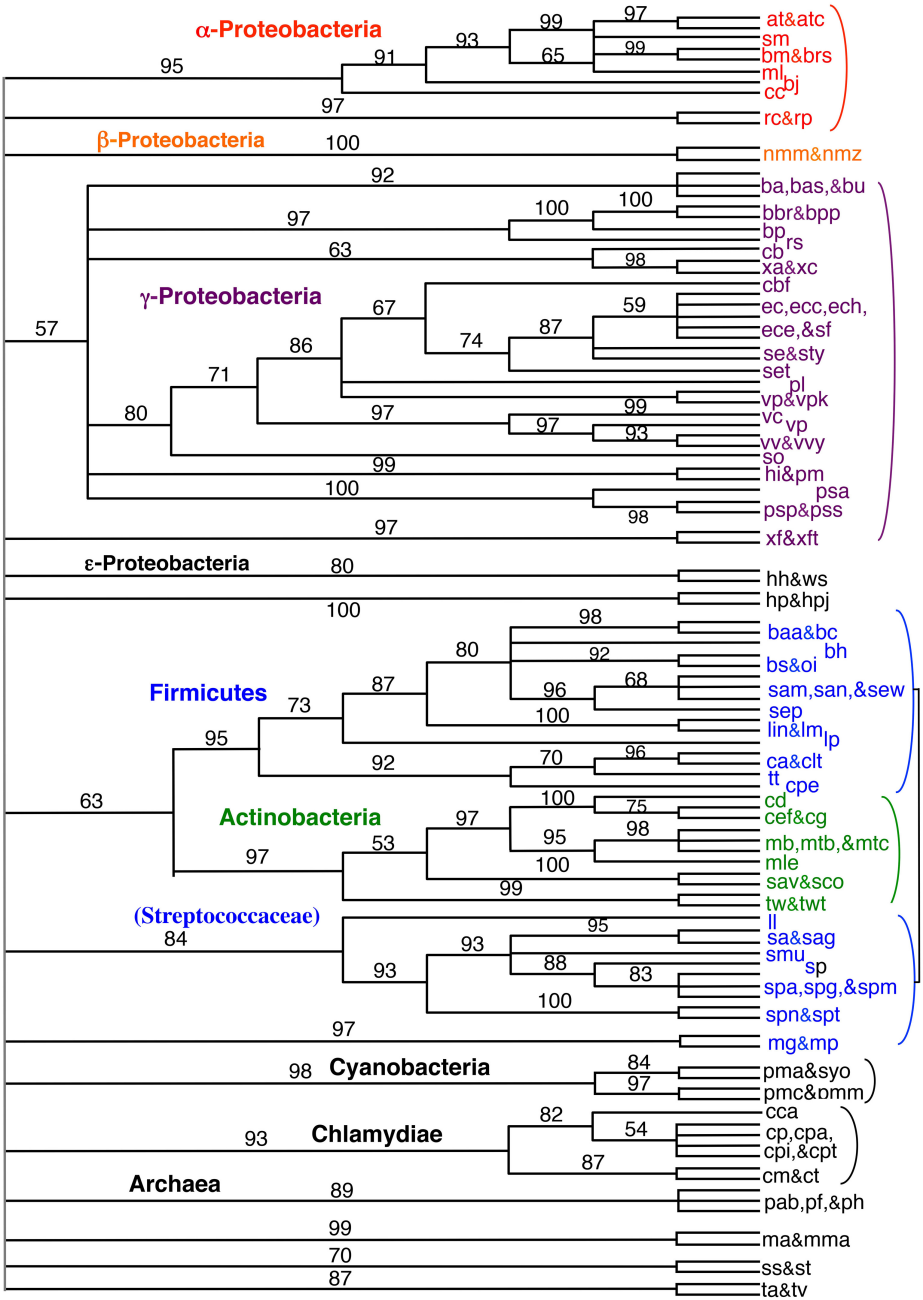
**“Bootstrap” NJ cladogram of the gene order distance tree shown in Figure 4**. Each node shows the number of times that node appears in 100 replicate trees each using gene order distances based on 100,000 iterations. Select taxonomic groups are labeled with the same color scheme as used in later figures.

Encouraged by the strong correlation between our distributed gene order distances and those of rRNA, we proceeded to build gene order-based neighbor joining (NJ) trees. Figure 4 shows a phylogram based on all 143 taxa, although unresolved single taxon are not shown for clarity. Similarly, Figure 5 shows the bootstrap NJ tree when all taxa are included. The results show that major bacterial taxonomic groups are mostly correctly clustered. With the largest number of resolved taxa, the γ-Proteobacteria shows the best resolution, and has a branching order reasonably consistent with published reports (House et al., 2003; Brown and Volker, 2004; Belda et al., 2005). In contrast, Archaea are almost completely unresolved, with only generic-level similarity yielding clustering (e.g., *Pyrococcus*, *Methanosarcina*, *Sulfolobus*, and *Thermoplasma*). Interestingly, the Actinobacteria are resolved with high confidence as a sister group to the bulk of the Firmicutes (including *Bacillus*, *Clostridium*, *Lactobacillus*, *Listeria*, *Oceanobacillus*, *Staphylococcus*, and *Thermoanaerobacter*). In contrast, our bootstrap tree (Figure 5) does not cluster any of the proteobacterial classes together. Finding the Actinobacteria and Firmicutes united is interesting because they are the two phyla that comprise the “gram-positive bacteria.” While it has long been considered likely that the gram-positive bacteria are a monophyletic group, it has been to date remarkably hard to find supportive molecular sequence data, genetic or genomic (De Rijk et al., 1995; Olsen, 2001; Fu and Fu-Liu, 2002; Deeds et al., 2005).

Next, we tested if the small phylogenetic signal we found with gene order distance was due to the occasional sampling of ribosomal operons, despite the 5 gene exclusion zone. A detailed look at 100,000 randomly sampled gene sets revealed that sets with more than one ribosomal gene do not occur any more frequently for conserved order sets (20%) than non-conserved order sets (21%). Furthermore, there was very little difference in the percentage of each of the following cog-based (Tatusov et al., 2003) protein function categories between the two groups of sets (conserved vs. non-conserved): informational (24 vs. 26%), cellular (17 vs. 17%), metabolism (36 vs. 37%), poorly categorized (15 vs. 14%), no cog match (8 vs. 6%). This suggests that the signal is distributed across many different types of genes and is probably not due to unreliable “jackpot” effects of single operons. We also pruned our data set to remove all ribosomal genes. When this pruned data set was used for building a NJ tree, however, the resolution is reduced resulting in a topology where some well-established microbial phyla are intertwined. This new NJ result does unite the Actinobacteria and Firmicutes, but with very low confidence. Because the dataset with ribosomal genes removed does not fully reproduce the results shown in Figure 5, it remains a possibility that a notable proportion of the gene order signal is preserved in ribosomal genes, but that in addition the signal overall appears to be distributed across a variety of other gene functional categories. The most likely way to reconcile these apparently divergent conclusions is that the phylogenetic signal in gene order distance is small, and so, the removal of any class of genes (including ribosomal operons) appreciably reduces the robustness of the results.

### Additional gene order tree building starting from 143 taxa

To complement our NJ tree building exercise using all 143 taxa, we aimed to address the fundamental problem that only a portion of our 10,153 pair wise gene order distances were significant and should be useful for tree building. The inclusion of genome pairs that are too diverged with respect to their gene order has the potential to alter the observed position of other taxa on a tree. This concern is not unique to gene order data. It has long been known that with sequence data, the uncertainty on an estimated distance goes up greatly with the magnitude of the divergence (Kimura and Ohta, 1972). Gene order data though provide a dramatic example of how it can be difficult to accurately estimate divergence when organisms are highly diverged. To minimize this problem, we proceeded with two additional tree studies.

**Figure 6:**
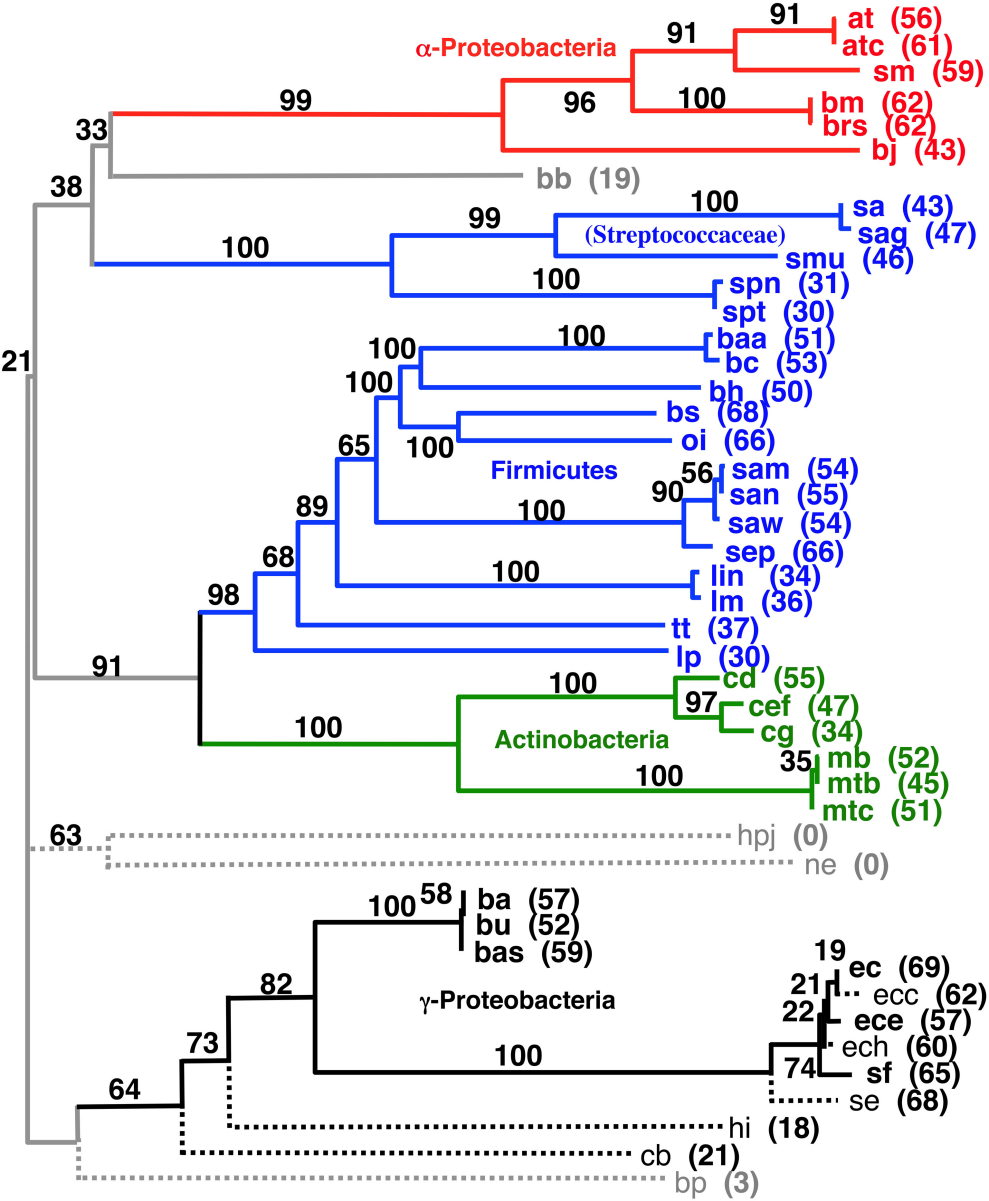
**Midpoint-rooted NJ phylogram based on our hierarchical tree building starting with 143 genomes (see Materials and Methods) using the same gene order data distances as the tree shown in Figure 4**. This resultant tree includes the most taxa during the initial round of clustering with solid lines and bold font. Taxa connected with dashed lines are those found to be compatible during a second round of single taxon addition. “Bootstrap values” shown are the number of times a node is found when NJ trees are formed using these taxa and the 100 replicate gene order distances. The values listed for individual taxa are the number of times that taxon is found in the biggest tree formed by the initial round of clustering when the 100 replicate gene order distances are used. Taxa not shown that were found 60 or more times in the largest tree after the initial clustering were: vc (70), cbf (67), vv (65), sty (63), set (61), and vp (60).

**Figure 7:**
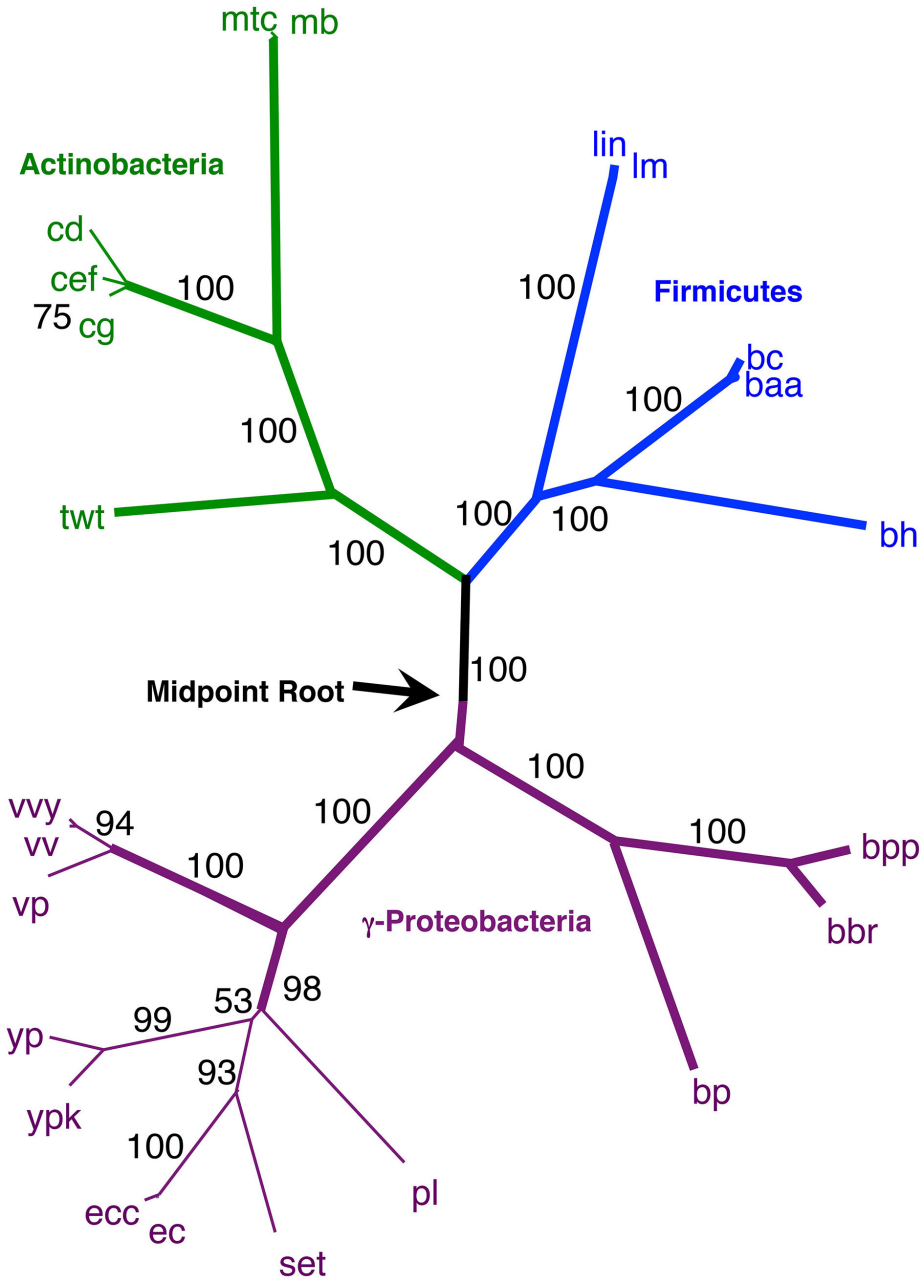
**NJ phylogram, starting with the 143 original taxa, limited to only the 23 taxa found in the agreement subtrees for the 100 replicate trees formed using iterations of six predicted orthologs**. Bold lines show the part of the tree that is found in all 18 agreement subtrees. “Bootstrap values” shown are the number of times a node is found when NJ trees are formed using these taxa and the 100 replicate gene order distances. Actinobacteria are shown in green and Firmicutes are shown in blue, while the γ-Proteobacteria shown in gray.

We tried developing a novel hierarchical and iterative tree building strategy (see Materials and Methods) based on the principle that our shorter distances are known with a higher degree of confidence than our larger distances. The goal of this approach is to provide a systematic and objective way to build a tree that includes as many of the pair wise gene order distances as possible without letting very distant (random) pairs adversely influence the observed phylogenetic positions of the more closely related taxa. Detailed results of this work are listed in the Supplemental Material. Figure 6 shows the largest tree formed starting this process with all 143 taxa. The tree in Figure 7 has 37 taxa added in the initial clustering process and another 8 taxa added during a second phase (single taxon addition). The result shows reasonable clusters representing the α-Proteobacteria, γ-Proteobacteria, Actinobacteria, and Frimicutes, plus a few other taxa from different poorly represented groups. The midpoint-rooted result again shows the Actinobacteria clustering with the bulk of the Firmicutes in a similar fashion to that shown in Figures 4, 5. The other well-supported trees from this analysis either also show such a clustering or do not contain taxa that can address the relationship between the Actinobacteria and Firmicutes. Also, observed in Figure 6 is the splitting of the Firmicutes into two groups with the Streptococcaceae (*Streptococcus* and *Lactococcus*) falling away from the bulk of the Firmicutes. A similar result was observed in the Figure 4, albeit with a different ultimate affinity for the Streptococcaceae. The inconsistent placing of this group on the trees found in Figures 4, 7, plus the unresolved placing of this group in Figure 5 and the exclusion of this group from Figure 6, collectively suggests that gene order is unable to confidently place this group on the tree—leaving it inconclusive to the question of whether they belong with the rest of the Firmicutes or even clustered with the gram-positive bacteria, but diverged prior to the Actinobacteria. However, the fact that *Lactobacillus* (labeled lp) is consistently clustered with the bulk of Firmicutes suggests that the Lactobacillales (which includes the Streptococcaceae) do belong with the rest of the Firmicutes, and therefore, in this case, the Streptococcaceae appear to be misplaced due to an artifact related to “long branch attraction.”

Secondly, using our original NJ trees, we identified the agreement subtrees for the 100 replicate NJ trees that had previously been constructed (and used for bootstrap analysis). Starting with the 100 trees, 18 agreement subtrees (each containing 18 taxa) were found. Together, the agreement subtrees contained a total of 23 different taxa. These 23 taxa were then used to build a new NJ tree (Figure 7) using the dataset constructed from all 10 million iterations. The result shows with high confidence three microbial groups—the Actinobacteria, the Firmicutes, and the γ-Proteobacteria. This pruned tree is the consistent core of the 100 replicate trees, and indicates that there is significant (but small) gene order conservation between these three taxonomic groups. When this tree is midpoint-rooted, the Actinobacteria and Firmicutes are united as sister groups with high confidence, which further suggests that the gram-positive bacteria might be monophyletic (as long as the assumptions inherent to midpoint-rooting are met). Based both of the conservative nature of this agreement substrees approach and the sensible results that it produces, we think that this is our best option for constructing a large gene order-based tree of prokaryotes.

### Gene order tree building starting from a more representative 172 taxa

**Figure 8:**
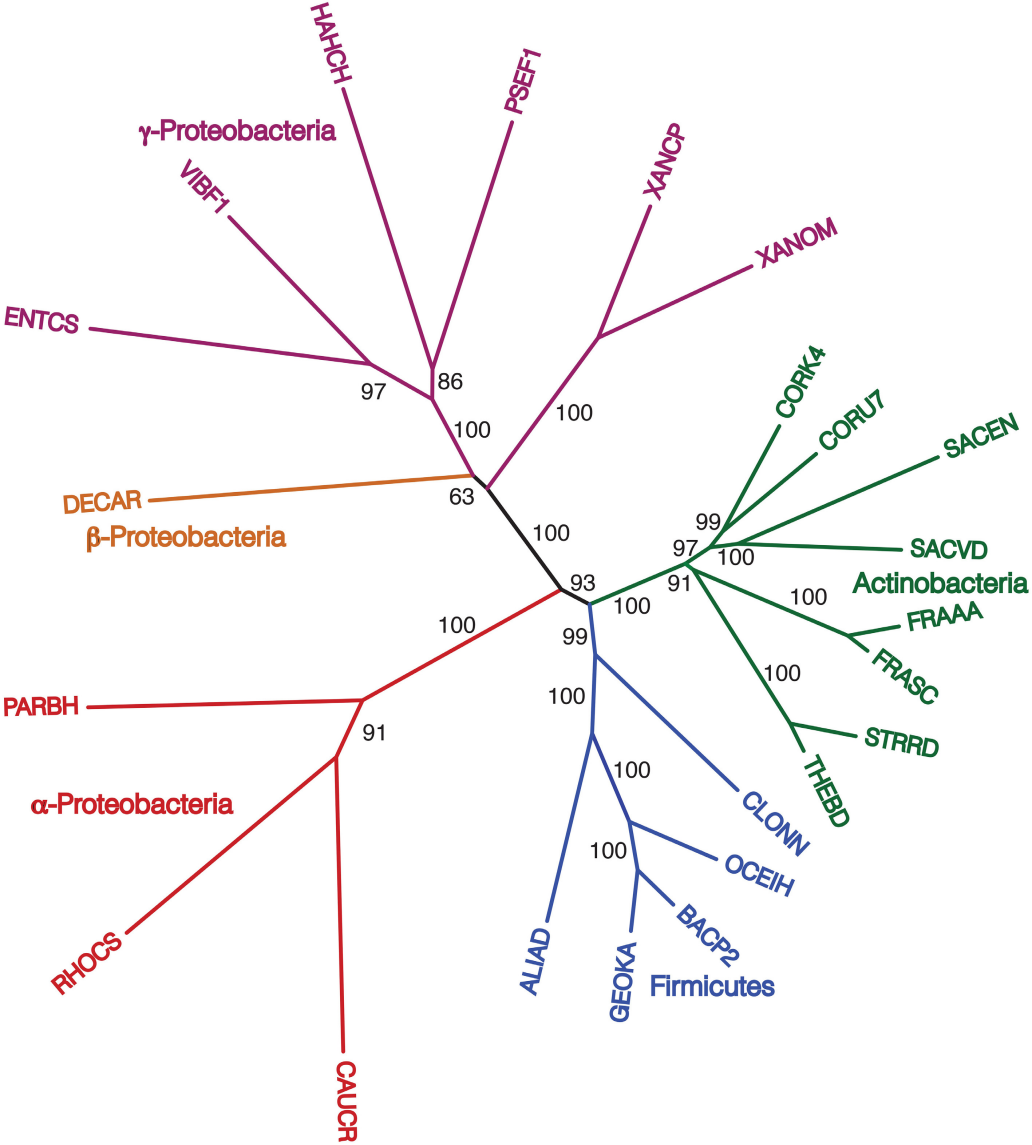
**NJ phylogram, starting with 172 representative taxa, limited to only the 23 taxa found in the agreement subtrees for the 100 replicate trees formed using iterations of six predicted orthologs**. “Bootstrap values” shown are the number of times a node is found when NJ trees are formed using these taxa and the 100 replicate gene order distances.

**Figure 9:**
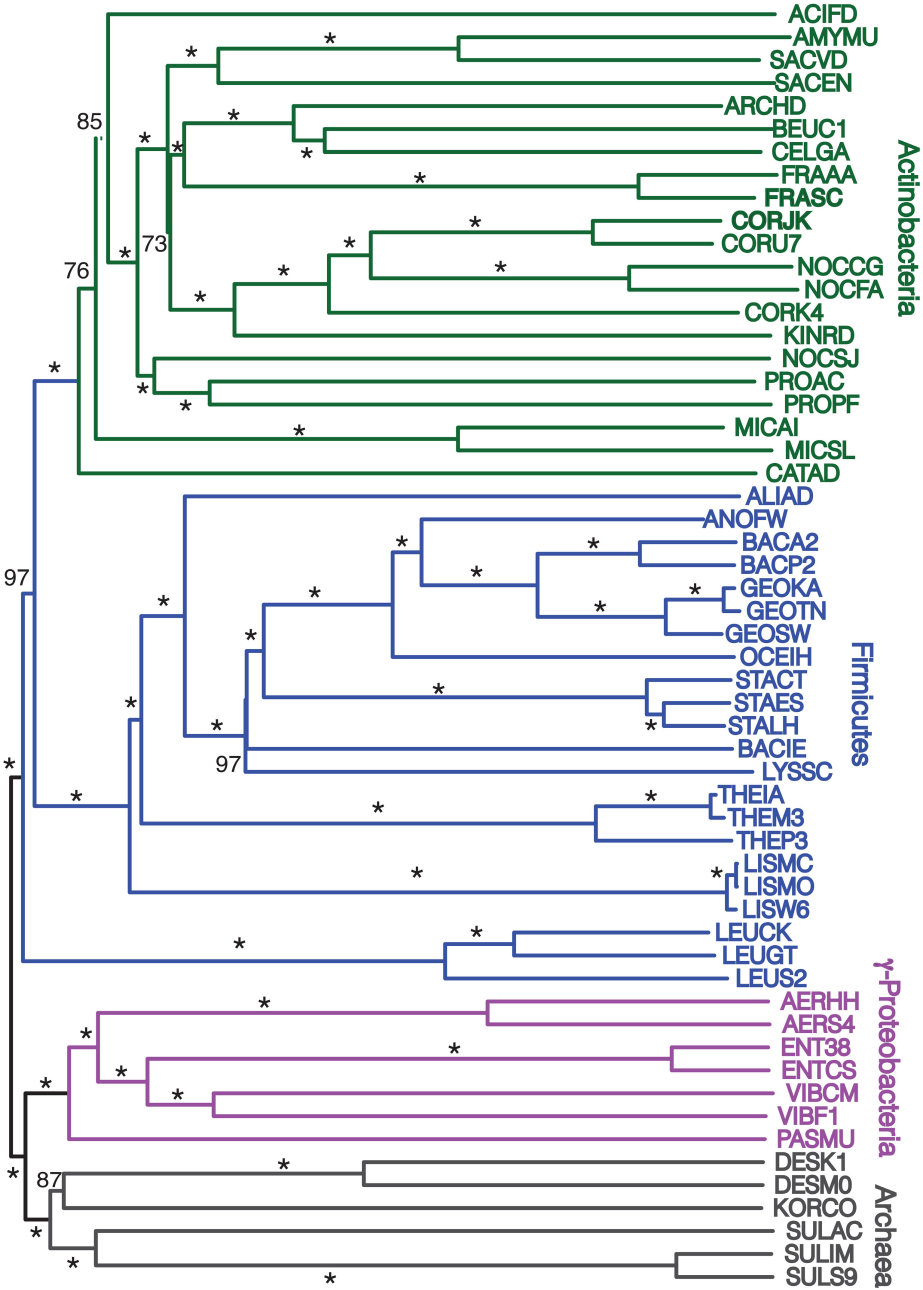
**Midpoint-rooted NJ phylogram, starting with 172 representative taxa, limited to only the 56 taxa found in the agreement subtrees for the 100 replicate trees formed using iterations of five predicted orthologs with the same distance equation as before, which ends up functionally equivalent to using D′ = −ln(1-D)**. “Bootstrap values” shown are the number of times a node is found when NJ trees are formed using these taxa and the 100 replicate gene order distances with ^*^ representing a bootstrap value of 100.

Finally, we repeated our agreement subtrees approach for our updated study of the relations of prokaryotes using 172 complete genomes (Table 3) better representing a wider-diversity of prokaryotes. With this fuller dataset, starting with 100 replicate NJ trees, the agreement subtree only contained 13 taxa. These 13 taxa were then used to build a NJ tree as before (Figure 7). As before, the resultant tree shows with high confidence that Actinobacteria and Firmicutes are sister groups (Figure 8). We also repeated this final analysis selecting five orthologs in the same order rather than six. This resulted in a summary agreement subtree with 56 taxa suggesting there is significantly more genomic gene order signal with five genes than with six. The 56 taxa tree (Figure 9), which now includes Archaea and Bacteria, again shows with high confidence that Actinobacteria and Firmicutes are sister groups forming a gram-positive clade (Figure 9). The midpoint rooting of this final tree (Figure 9) places Archaea as a sister group to the γ-Proteobacteria. At face value, this suggests there is a little more gene order conservation between the Archaea and the γ-Proteobacteria than with any other bacterial group. Gene order conservation between Archaea and the γ-Proteobacteria would argue against the “neomuran origin” for the archaea cell (Cavalier-Smith, 2002). A pairing of Archaea with the γ-Proteobacteria, though, should be taken with significant caution because the result is completely dependent on the midpoint rooting, which may incorrectly represent the history of these evolving groups. Using the Archaea as an outgroup, naturally would place the Proteobacteria with the other bacterial phylum represented. In either case, though, the tree supports the notion that the gram positive bacteria (Actinobacteria and Firmicutes) evolved once from a gram-negative relative. It is also notable that the genome-wide synteny tree of 89 microbes published by Shifman et al. (2014) also shows the Actinobacteria and Firmicutes united as sister groups, even though hat particular work used different genomes and a different approach to estimate gene order similarity across genomes.

### Implications of gene order conservation found

At this point, we can conclude that starting from a large number of genomes, we find, perhaps surprising, that there is some gene order conservation between a few major groups, namely Firmicutes, Actinobacteria, and Proteobacteria (Figures 4–9) and less robustly the Archaea and Proteobacteria (Figure 9). Comparison of genomes from closely related species reveals that inversions are quite common. Large inversions involving up to half of the genome are found frequently between closely related species (e.g., within the *Pyrococcus* genus, Zivanovic et al. (2002), within the *Yersinia* genus, Darling et al., 2008). Given this potentially very rapid rate of divergence in gene order, it is surprising to find residual phylogenetic signal still uniting such distant groups as the Actinobacteria and the Firmicutes. However, while large inversions are common, they are not random in their distribution. For example inversions that disrupt the symmetry of the replicons are frequently not tolerated (Eisen et al., 2000; Zivanovic et al., 2002; Darling et al., 2008). Thus, the rapid changes may be restricted in their range leaving large portions of the genome with potentially conserved gene order over large time scales.

Taken together, our results suggest that the Actinobacteria is a sister group to the Firmicutes, which in turn implies a single origin for the gram-positive cell. Since the first few whole genome sequences were published, some genomic trees have failed to unite these groups (Brown et al., 2001; Fu and Fu-Liu, 2002; Korbel et al., 2002), while others have found weak support for the pairing (House et al., 2003) or have found the pairing under a subset of conditions tried (Deeds et al., 2005). There are three possible disparate causes for these results. First, it is possible that the gram-positive cell has evolved more than once in Earth history. In particular, it has been suggested that *Mycobacterium* may have a close relationship to gram-negative bacteria (Fu and Fu-Liu, 2002). Second, it has been hypothesized that gram-positive bacteria are more primitive than gram-negative bacteria (Gupta, 1998; Errington, 2013). Third, some researchers are of the opinion that the failure of genomic methods to unite the gram-positive bacteria together indicates that genomic methods are still inadequate to address this relationship (Olsen, 2001), and that ultimately, we will find that the gram-positive bacteria could be united as a monophyletic group. In particular, the strong similarity in the structure of the cell walls of Firmicutes and Actinobacteria argues for a single origin. The gram-positive cell type, found in both Firmicutes and Actinbacteria, consists of thick layers of peptioglycan with teichoic acids and a single membrane. Gram-negative bacteria have a thin peptidoglycan layer, lack teichoic acid, and have a second outer membrane with lipopolysaccharides.

Considering that our gene order analyses have consistently produced trees with the Actinobacteria united with the bulk of the Firmicutes to the exclusion of other bacterial groups (mostly the Proteobacteria), our results support the uniting of these groups and argue against multiple origins for the gram-positive cell type. The strongest evidence against a strict monophyletic pairing of the Firmicutes with the Actinobacteria comes from the (unrooted) phylogenetic analysis of 31 concatenated bacterial genes (Wu et al., 2009) and 24 concatenated bacterial genes (Lang et al., 2013), which appear to support a mostly gram-positive clade of Firmicutes, Actinobacteria, Chloroflexi, and Cyanobacteria. Incidentally, Lang et al. (2013) also show the Tenericutes as part of the Firmicutes. At present, we cannot rule out such a larger (primarily) gram-positive clade because it is possible that other phyla (like the Tenericutes) will be included within our Firmicutes/Actinobacteria cluster when taxa sampling increases for gene order studies. Generally, one can argue that because several of our trees (those restricted to agreement subtrees) do not include any taxa from bacterial groups other than the Proteobacteria, we cannot rule out the possibility that one of the other phyla, such as the Cyanobacteria, would break up our Firmicutes/Actinobacteria clade. However, such reasoning does requires the taxa within such a phyla to have all scrambled their gene order to the point to which they show no affinity to either the Firmicutes or Actinobacteria in spite of their supposed closer affinity. Our results though still show a uniquely strong conservation of gene order between the Firmicutes and Actinobacteria. We, therefore, feel that our results are indicative of a tree of life in which most other bacteria phyla diverged prior to the base of a gram-positive cluster (either Firmicutes/Actinobacteria or a larger similar clade). This interpretation in turn implies a single origin for the gram-positive cell. Our results also indicate that gene order of certain genomes are phylogenetically informative at both low and high taxonomic levels, but that for many other genomes gene order is not conserved for a long time.

### Conflict of interest statement

The authors declare that the research was conducted in the absence of any commercial or financial relationships that could be construed as a potential conflict of interest.
